# Exploring the threshold premium for viable community based health insurance schemes in Nigeria

**DOI:** 10.1186/s13104-016-2185-1

**Published:** 2016-08-02

**Authors:** Emeka Ihechi Udeh, Obinna Emmanuel Onwujekwe, David Ayobami Adewole, Chima Ariel Onoka

**Affiliations:** 1 Department of Surgery, Faculty of Medicine, College of Medicine, University of Nigeria, Nsukka, Nigeria; 2 Department of Health Administration and Management, Faculty of Health Sciences and Technology, College of Medicine, University of Nigeria, Nsukka, Nigeria; 3 Department of Pharmacology and Therapeutics, Faculty of Medicine, College of Medicine, University of Nigeria, Nsukka, Nigeria; 4 Department of Community Medicine, Bowen University of Ibadan, Ibadan, Nigeria; 5 Department of Community Medicine, Faculty of Medicine, College of Medicine, University of Nigeria, Nsukka, Nigeria

**Keywords:** Threshold premium, Community based health insurance scheme, National health insurance scheme

## Abstract

**Background:**

The national health insurance scheme of Nigeria recently proposed a national premium for community based insurance scheme. This study determined the capacity of households in the rural and urban areas in Nigeria to pay for the premium and different hypothetical health insurance schemes namely national health insurance scheme, national urban health insurance scheme, national rural health insurance scheme and regional health insurance schemes. It determined the likely impact of different premiums on membership across socio-economic status quintiles, and then determined the threshold premium affordable to rural and urban households.

**Results:**

The results show that the mean capacity to pay for the households in different regions ranged from US$194 ± 100 to US$986 ± 907. The threshold premiums of the national health insurance scheme, urban national health insurance and rural health insurance schemes were US$66, US$154 and US$53 respectively.

**Conclusions:**

Overall, the threshold premium for rural national health insurance scheme and national health insurance schemes were affordable to the lowest socio economic group. Hence, it is recommended that threshold premium for rural national health insurance scheme be adopted as the maximum premium not to be exceeded in the proposed national health insurance scheme.

## Background

The essence of health insurance is to mobilise healthcare resources from citizens through a prepayment system and to protect individuals and households from catastrophic health expenditures, in order to enhance population coverage with quality health services.

Although, health insurance confers financial protection [1], complementary risk protection is predicated on the extent of co-payment for access of health services; the depth of the benefit package and the perception of the quality of service provision [2–4].

Many low income countries are compelled to rely on community based health insurance (CBHI) to extend financial risk protection to citizens [4]. This occurs because most of these countries lack robust taxation systems and are plagued by high unemployment rates that make it difficult to adopt either general tax based health insurance or social health insurance as vehicle to universal coverage [5].

Community based health insurance schemes depend on the voluntary contribution of members. However, the financial pool achieved by such schemes is usually small, and often inadequate to ensure the provision of the benefits included in the scheme, and the payments that need to be made to providers. Having voluntary contributions also creates the room for the healthier population to opt out of schemes, and for wealthier groups to aggregate within schemes that offer more comprehensive services compared to those populated by poorer people, which thus compromises the cross-subsidization objective of insurance pools.

The flat nature of contributions also mean that redistribution of resources is not achieved. These factors create the situation that CBHI schemes are unlikely to guarantee financial pools that provide adequate financial risk protection for members, except if they are complemented with subsidies, reinsurance or are merged [5, 6].

Achieving financial sustainability and high enrolment in CBHI schemes considerably depends on the level of the CBHI premium. Low CBHI premium usually result in unsustainable schemes which ultimately collapse, with the attendant effect that members end up depending on out-of-pocket payments for healthcare. Conversely, high CBHI premiums may not be affordable. An upward review of premium in CBHI usually stimulates de-enrolment of members, and may disproportionately affect the poor members of the scheme [7].

One way of determining the readiness of a population to embrace an insurance premium, is through willingness to pay studies [8–14]. Most times, premiums generated from WTP in low income countries are short of what can financially sustain such insurance schemes based on such premiums [15].

Since willingness to pay is also linked with affordability of the premium, some authors have also modelled hypothetical scenarios that analysed affordability of premiums at different thresholds. Schmidt, Mayindo and Kalk [15] in their study of determining how much Rwanda’s population should pay for CBHI, modelled various and increasing household membership fees in order to predict the percentage of the population excluded from CBHI. Based on the fee exceeding monthly income of the respective stratum, they determined the threshold premium (TP).

In this study, the threshold premium (TP) is being evaluated, which is, the maximum level of premium that a given proportion of the population covered by the scheme will be able to afford based on a pre-determined capacity to pay income. This study is therefore, meant to fill the existing gap in knowledge with respect to affordable premium and sustainable CBHI based on the affordable premium. The paper provides new knowledge on the threshold premium for a viable social health insurance scheme for the informal sector in Nigeria.

### Conceptual framework

This study analyses affordability of CBHI premiums by determining households’ capacity to pay for a set of flat rate premiums of hypothetical CBHI schemes, and also models the likely impact of such premiums on membership of these schemes.

The capacity to pay (CP), is defined here as 40 % of the annual non-food expenditure of the households in line with the recommendation of the World Health Organisation [16], and is estimated for households categorised in various geographic groups, and socio-economic status quintiles. The capacity to pay amount is equivalent to the amount, if exceeded, is regarded to result to catastrophic medical expenditure [16–19]. For this study, catastrophic medical expenditure is said to occur when a “household must reduce its expenditures over a period of time to cope with health costs, although no consensus is available on the threshold proportion of household expenditure” [16].

Though there has not been consensus on what percentage of non-food expenditure should be regarded as catastrophic medical expenditure, a range of values have been proposed by experts in literature. (I.e. 5–40 %) [16–22]. In Nigeria, private funding for Medicare is more than 90 % [23]. More than 70 % of the population live below $1 a day [24] and prepayment mechanism for pooling risk is lacking [23]. Adopting the threshold of 40 % for catastrophic medical expenditure showed that 14.8 % of households studied in South Eastern Nigeria [24] and 15 % of households studied in urban South Eastern Nigeria were affected [25]. Both studies showed that the lowest socio economic quintile were most affected. Since, most studies done in Nigeria on catastrophic medical expenditure based on the maximum value of 40 % showed that the lowest SES was affected, we adopted this threshold in determining capacity to pay [26]. Non-food consumption expenditure is used as proxy to income because it is sensitive; information on income are not easily and accurately declared in African countries [23].

Achieving total health insurance coverage should be the objective of policy makers and the government. In our context whereby health insurance for the informal sector has not been properly established, we assumed that as part of the initial protocol for implementation, government will be willing to cover at least 80 % of the population. Since government as part of her civic responsibility will make provision in the budget annually for the health sector,

We assumed that money generated by insurance schemes will be augmented by the budget of Ministry of Health.

## Methods

### Study setting, data and sampling approach

Data for this study is a revised general household survey panel (GHS-Panel) fielded by the National bureau of statistics in conjunction with the World bank in Nigeria between 2010 and 2011 [16].

The sample is designed to be representative at the national level as well as at the zonal (urban and rural) level. The sample is a two-stage probability sample:

*First stage* The Primary Sampling Units (PSUs) were the Enumeration Areas (EAs). These were selected based on probability proportional to size (PPS) of the total EAs in each state and Federal Capital Territory (FCT), Abuja and the total households listed in those EAs. A total of 500 EAs were selected using this method.

*Second stage* The second stage was the selection of households. Households were selected randomly using the systematic selection of ten (10) households per EA. This involved obtaining the total number of households listed in a particular EA, and then calculating a sampling interval (SI) by dividing the total households listed by ten (10).

The next step was to generate a random start ‘r’ from the table of random numbers which stands as the 1st selection. Consecutive selection of households was obtained by adding the sampling interval to the random start. Determination of the sample size at the household level was based on the experience gained from previous rounds of the GHS, in which 10 households per EA are usually selected and give robust estimates.

In all, 500 clusters/EAs were canvassed and 5000 households were interviewed. These samples were proportionally selected in the states such that different states had different samples sizes. The distribution of the samples are shown in Table 1 below which shows the size of the sample in each state, by geopolitical zone and urban/rural break-out. Households were not selected using replacement; thus the final number of household interviewed was slightly less than the 5000 eligible for interviewing. A total of 27,993 household members were interviewed. On the average about 4–6 all adult members per house hold. Thus the final number of households with data in both points of time (post-planting and post-harvest) is 4851 for a non-response rate of 0.3 percent.

**Table 1:** Distribution of final sample of 500 EAs and 5000 households for panel survey by State, urban and rural sectors, within each zone

	State	Total		Urban		Rural	
		No. EAs	No. Hhs	No. EAs	No. Hhs	No. Hhs	No. EAs
North central zone	Benue	16	160	2	20	14	140
	Kogi	12	120	4	40	8	80
	Kwara	12	120	6	60	6	60
	Nassarawa	7	70	1	10	6	60
	Niger	18	180	4	40	14	140
	Plateau	11	110	2	20	9	90
	FCT Abuja	4	40	3	30	1	10
North-east zone	Adamawa	12	120	1	10	11	110
	Bauchi	17	170	3	30	14	140
	Borno	21	210	5	50	16	160
	Gombe	8	80	1	10	7	70
	Taraba	9	90	0	0	9	90
	Yobe	13	130	3	30	10	100
North-west zone	Jigawa	13	130	2	20	11	110
	Kaduna	12	120	4	40	8	80
	Kano	20	200	3	30	17	170
	Katsina	18	180	3	30	15	150
	Kebbi	10	100	1	10	9	90
	Sokoto	8	80	2	20	6	60
	Zamfara	9	90	2	20	7	70
South-east zone	Abia	11	110	4	40	7	70
	Anambra	22	220	12	120	10	100
	Ebonyi	14	140	1	10	13	130
	Enugu	14	140	3	30	11	110
	Imo	19	190	2	20	17	170
South–south zone	Akwa-ibom	15	150	4	40	11	110
	Bayelsa	7	70	1	10	6	60
	Cross River	13	130	3	30	10	100
	Delta	14	140	4	40	10	100
	Edo	10	100	5	50	5	50
	Rivers	21	210	8	80	13	130
South-west zone	Ekiti	8	80	6	60	2	20
	Lagos	17	170	16	160	1	10
	Ogun	11	110	7	70	4	40
	Ondo	13	130	6	60	7	70
	Osun	18	180	14	140	4	40
	Oyo	23	230	15	150	8	80

*EAs* enumeration areas; *Hhs* households

The survey covered a wide range of topics which were collected via three different questionnaires administered to the household and the community. These were a household questionnaire, an agricultural questionnaire and a community questionnaire. The questionnaire obtained information on socio-economic and demographic characteristics of households, labour and use of time, expenditure on education, health, food and non-food items, household non-farm income-generating activities, assets, and use of information and communication technology.

### Study design

The study was designed to present various scenario for estimation of affordability of premiums, and to use information from the data sources to model the threshold premium. It also modelled the capacity to pay premium of households based on 40 % of their annual total non-food expenditure (Table 2).

**Table 2:** An overview of different models of hypothetical schemes

Scenarios	Hypothetical health insurance schemes	Coverage
2	National health insurance scheme	The whole population
2A	National rural health insurance scheme	The whole rural population
2B	National urban health insurance scheme	The whole urban population
3	Regional health insurance schemes	The six different regions

For this study we modelled the effects of premium on the populations’ CP, for different rolled-out national and regional health insurance schemes. Several scenarios were considered. The national and regional schemes were considered, since most WTP studies in Nigeria were conducted at state levels and so many states in the Northern region have not had any WTP study conducted for them.

*Scenario 1* The proportion of the population able to afford different models of CBHI premiums (generated in the multiples of five, ranging from US$0–US$140) based on their ‘capacity to pay’ income. The choice of the range of premium was based on findings from WTP studies in Nigeria [12, 14, 17].

*Scenario 2* A hypothetical national insurance scheme to cover the whole population in the informal sector was postulated. It was further differentiated into:

National rural insurance scheme: This scheme should cover only the rural population in the nation.

National urban insurance scheme: This should cover only the urban population in the nation.

*Scenario 3* Hypothetical regional health insurance schemes to cover the six different regions in Nigeria were considered.

These include:

The North-central insurance scheme: Such a scheme is defined as covering the population in the North-central region. Therefore, TP would be based on the CP of the population within the region.

The North-east insurance scheme: Such a scheme is defined as covering the population in the North-east region. Therefore, TP would be based on the CP of the population within the region.

The North-west insurance scheme: Such a scheme is defined as covering the population in the North-west region. Therefore, TP would be based on the CP of the population within the region.

The South-east insurance scheme: Such a scheme is defined as covering the population in the South-east region. Therefore, TP would be based on the CP of the population within the region.

The South-west scheme: Such a scheme is defined as covering the population in the South-west region. Therefore, TP would be based on the CP of the population within the region.

The South-south insurance scheme: Such a scheme is defined as covering the population in the South-south region. Therefore, TP would be based on the CP of the population within the region.

*Scenario 4* Hypothetical regional schemes divided into North and South insurance schemes with a political bias in favour of the north. The political biased North insurance scheme will cover all the three northern geopolitical zones while the politically biased south insurance scheme will cover all the three southern geo-political zones. TP would be based on the CP of the populations covered by these schemes. The bias in this scheme is that effort is being made to cover a larger proportion of the population in the North which is considered to have a greater population of population in the lowest socio economic quintile at the expense of the southern regions.

Under scenario I: the threshold premium is assumed to be:

P_1_ = threshold premium that will be afforded by 80 % of the population in the informal sector.

Under scenario 2: the threshold premium is assumed to be:

P_2_ = threshold premium that will be afforded by 80 % of the population within the urban and rural areas respectively.

Under scenario 3: the threshold premium is assumed to be:

P_3_ = threshold premium that will be afforded by 80 % of the population within each region.

Under scenario 4: the threshold premium is: is assumed to be:

P_4_ = threshold premium that will be afforded by 90 and 70 % of the population within the North and South regions of Nigeria respective

### Data analysis

Calculating Insurance coverage. Assuming that the whole population of Nigeria was P and the population for the six regions were P1, P2, P3, P4, P5 and P6 respectively.

Also, if government seeks to cover 80 % of the population in order to achieve universal coverage, the population covered will be expressed mathematically as:

$0.8p = x1p1 + x2p2 + x3p3 + x4p4 + x5p5 + x6p6;$ where x_i_ represents the proportion of population from region i covered by the insurance. In a simplest and most equal form, which we model first (i.e. scenario 1—see above), all xi are equal to 80 %. But alternative coverage options are possible where coverage will be different from one region to another.

Calculating capacity to pay income. The capacity to pay of households is defined as:

$$CP = ExpNF \times 40\,\%$$

where ExpNF is the total expenditures spent by a household on non-food products.

Based on the CP, the households in the study were arranged in descending order and the percentile with confidence interval calculated. The 20th percentile was used except in the case of politically- biased North and South schemes in which 10th and 30th percentile were used respectively. The values gotten following the calculation of percentile using the 20th percentile (10th and 30th percentiles for politically biased schemes) were regarded as the TPs for the different schemes in question.

Calculating insurance financial sustainability. Maximum income generated by Insurance scheme is defined as:

$$Max - Tinc = TP \times HH$$

where TP is the threshold premium per household; HH is number of households covered by scheme.

Therefore income generated per person by insurance scheme is defined as:

$$Iper = Max - Tinc/Px$$

where P _x_ is the total population covered by the scheme.

An assumption was made that the money generated from each scheme would be augmented by the annual funds transfer to the ministry of health (MOH) by the federal government.

Therefore, Revenue budgeted per person by MOH is defined as:

$$Rper = Trev/P$$

where T _rev_ is the total budget to MOH and P is the total population of the nation.

Therefore, total income generated per person by each scheme following funds transfer from MOH is defined as:

$$Tincome = Iper + Rper$$

where I _per_ is income generated per person by insurance scheme and R _per_ is revenue budgeted per person by MOH.

Data analysis was done using STATA 12 and Microsoft excel 2007 spread sheet

Currency Conversion rate used in the study: 155.73 Naira = US$ 1 [18].

## Results

### Descriptive characteristics of all respondents

About 68.31 % of the respondents in this study were living in rural area. Half of these respondents (50.1 %) were women.

The average age of the respondents were 23 years for both urban and rural population. The average annual total expenditure of households was 486,867 Naira (US$ 3126). Maximum annual average expenditure on health constitutes 6 and 18 % of average total and non-food expenditures respectively.

### Expendable income for population strata

Table 3 summarizes mean capacity to pay categorized in quintiles used in analysis. Average CP ranged from 12,267–124,576 Naira (US$79–US$800). The highest CP was seen in 5th quintile of South-west zone while the lowest was in 1st quintile of North-west zone. The CP increased along quintiles for all zones. There appears to be variation in statistical difference in average CP between urban and rural sectors in the different zones. The north central and North West zones showed consistently, a significant statistical difference in average CP between urban and rural sectors across quintiles.

**Table 3:** The capacity to pay across zones categorized in quintiles

SES quintiles	Mean capacity to pay in Naira categorized in SES quintiles ± SD				
	1	2	3	4	5
North-central					
A	30,271 ± 15,577	41,039 ± 25,855	50,911 ± 32,154	70,288 ± 49,888	111,032 ± 101,971
B	101,971 ± 12,683	24,123 ± 20,470	30,488 ± 26,157	45,772 ± 45,092	78,297 ± 91,154
P	<0.001*	0.001*	0.001*	0.002*	0.010*
North-east					
A	42,757 ± 53,440	30,248 ± 19,899	54,103 ± 40,784	57,864 ± 49,689	122,532 ± 185,815
B	13,786 ± 11,972	21,814 ± 20,334	37,577 ± 41,139	42,179 ± 41,609	74,570 ± 163,508
P	<0.001*	0.070	0.03	0.11	0.16
North west					
A	22,940 ± 13,782	33,551 ± 17,621	44,386 ± 35,952	52,455 ± 39,918	106,459 ± 107,559
B	11,368 ± 9244	17,844 ± 14,364	23,657 ± 20,842	33,355 ± 32,702	79,712 ± 144,794
P	<0.001*	<0.001*	<0.001*	0.005*	0.200
South east					
A	18,857 ± 14,946	24,784 ± 19,492	42,265 ± 37,335	56,996 ± 38,748	124,453 ± 114,897
B	16,106 ± 11,394	27,407 ± 18,227	32,613 ± 30,002	45,717 ± 38,821	68,182 ± 76,704
P	0.640	0.560	0.190	0.070	<0.001*
South–south					
A	22,388 ± 14,326	37,875 ± 23,783	40,057 ± 29,574	57,697 ± 41,776	142,574 ± 175,612
B	15,035 ± 11,826	29,522 ± 28,056	25,055 ± 21,448	37,964 ± 42,617	59,107 ± 130,511
P	0.060	0.130	0.005*	0.003*	<0.001*
South west					
A	24,785 ± 13,106	27,670 ± 16,954	53,637 ± 34,087	82,176 ± 55,001	153,558 ± 141,306
B	18,671 ± 12,859	26,345 ± 19,631	41,802 ± 27,930	55,811 ± 45,847	88,019 ± 101,698
P	0.140	0.820	0.08	<0.001*	<0.001*

*A* urban sector; *B* rural sector; *P* *p* value

* P < 0.05 statistically significant

### Modelling the likely impact of different CBHI fee structure on capacity to pay premium across quintiles

**Fig. 1:**
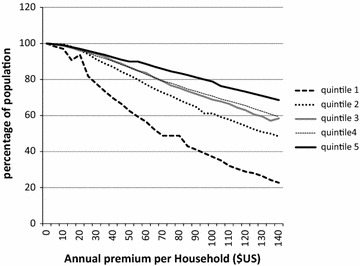
Proportion of population who could afford CBHI premium level

Figure 1 shows the linear relationship between premium levels and ability to afford premium. Sixty percent of the population could afford premium at US$60 annual premium irrespective of the quintile. However, beyond that level, there was a marked reduction of the population within the 1st quintile who could afford premium. Only 22 % of 1st quintile could afford premium of US$140 compared to 69 % in 5Th quintile.

At premium US$25, 80 % of the population within 1st quintile had the capacity to afford. There is an interesting overlap between 3rd and 4th quintile with respect to CP. Of all quintile the 1st quintile was affected most when premium exceeded US$60 (marked divergence of line in the graph).

### The threshold premium for rural and urban households in different Health Insurance Schemes

Table 4 shows that the 95 % confidence interval of all predicted TPs was within a narrow range and above zero. This gave the range that truly contains the actual population value. The politically biased scheme in favour of the northern part of the country generated a TP of 4562 Naira (US$29). This is about five times less than the TP of the politically biased south health insurance scheme (US$144).

**Table 4:** The threshold premium that households would be able to pay for the schemes

Health insurance schemes	Threshold premium (US$)	95 % confidence interval	Percentage of population included (%)	Number of observations (used for calculation)
(US$)				
North-central regional	9694 (62)	8526–11,096	80	788
North-east regional	7979 (51)	6894–8684	80	793
North-west regional	6804 (44)	6230–7700	80	892
South-east regional	11,222 (72)	9,9745–13,903	80	792
South-west regional	18,815 (120.82)	16,326–21,316	80	754
South-south regional	16,908 (109)	15,103–18,232	80	1528
National urban	23,905 (154)	21,483–25,693	80	1528
National rural	8218 (53)	7757–8699	80	3371
National	10,303 (66)	9862–10,851	80	4845
Politically-biased (north)	4562.48 (29)	4200–4873	90	2473
Politically-biased (south)	22,474 (144)	21,281–23,920	70	2372

155.73 Naira = US$1 [18]

It is of interest to note that though most regional schemes gave rise to TPs less than US$75, it was not same for both the south-west and south–south regional schemes which exceeded TPs of US$108.

Moreover, the national urban insurance scheme TP was three times higher than the national rural TP. The TP for the national insurance scheme appears to be equivalent to the least regional scheme TPs and the rural national insurance TP.

**Fig. 2:**
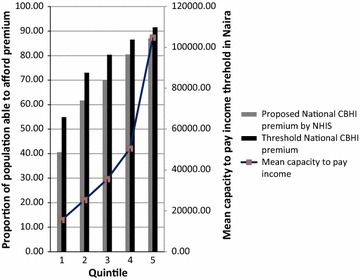
Proportion of the population who can afford premium based on national scheme categorized in quintiles

However, it is relevant to note the impact of these TPs on the proportion of the population able to afford them across quintiles. Figure 2 shows that 55 % of the population in 1st quintile could afford the TPs for the national insurance scheme compared to 91.5 % of those in the 5th quintile. Compared to the proposed national premium by NHIS, more individuals in the 1st quintile were able to afford the TP than the proposed national premium. Though this difference reflected across all quintiles, it was more marked in the 1st quintile.

There was a gradual rise in the mean CP across the quintiles. The population in the 1st quintile had the lowest mean CP which was below 20,000 naira per annum.

**Fig. 3:**
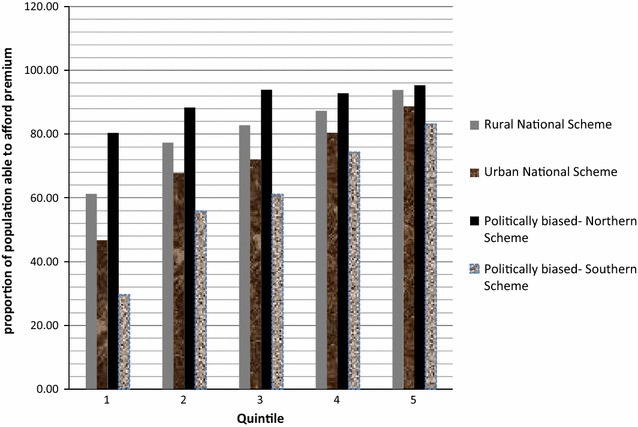
Proportion of the population who can afford premium based on National-based schemes categorized in socio-economic quintiles

Figure 3 shows that the proportion of the population in the 1st quintile that could afford TP was least in the politically biased south scheme. The TP of the politically biased north scheme (80 %) was the most affordable by those in the 1st quintile followed by the rural national scheme (60 %).

**Fig. 4:**
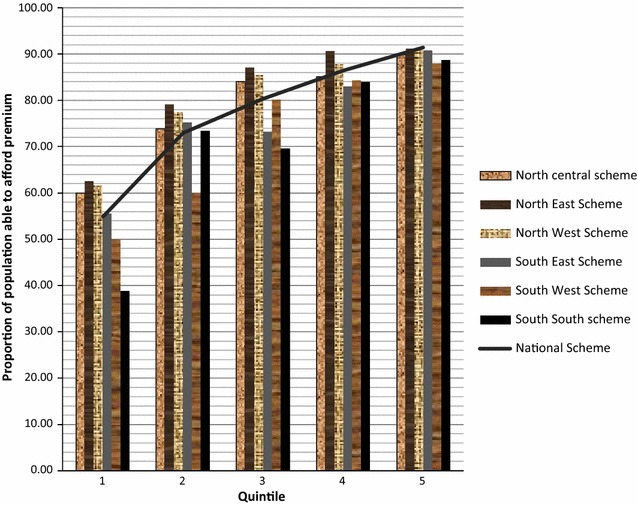
Proportion of the population who can afford premium based on regional health schemes categorized in socio-economic quintiles

Figure 4 shows the proportion of the population that can afford TP based on regional health insurance schemes categorized in socio-economic quintiles. The proportion of the population in 1st quintile able to afford the TP of the South-south regional scheme was the least (39 %). Three regional TPs were affordable by 60 % of the population within the 1st quintile namely north-central, north-east and north-west. Importantly, it should be noted that the proportion of the population within the 1st quintile who could afford TPs of regional schemes in the south were not up to 60 %.

Compared with the TP of the national scheme, only three regional schemes had similar effect on the population in the lowest quintiles ability to afford while the rest had worse effect. The variation in effect of the different regional schemes compared to the national scheme implies that the regional schemes may be more favourable to the lowest quintiles in some regions than in others; raising the issue of equity.

### The financial sustainability of the various schemes based on affordability threshold premium

Table 5 shows the financial sustainability of the various schemes based on TPs and funds transferred to ministry of health by the Federal Government of Nigeria. The approved budget for the ministry of health in 2011 was 235,866,483,244 Naira (US$1,742,624,949) [19]. With an estimated population of 150,589,000 [20], the budget per capita is about US$10. Therefore, the supplementary revenue that will be generated by each scheme based on the assumption of six persons per household [12] is shown in the Table 4 below:

**Table 5:** Showing income generated per individual for each scheme following Ministry of health funds transfer

Schemes	Population	Threshold affordable premium (US$)	income generated per person (US$)	Budget per capita (MOH)
Urban	50,147,000	154	36	10
Rural	100,442,000	53	19	10
Political-north	81,237,000	29	15	10
Political-south	69,353,000	144	34	10
National	150,589,000	66	21	10
Regional schemes				
North central	21,971,000	62	20	10
North east	20,353,000	51	19	10
North west	38,913,000	44	17	10
South east	17,430,000	72	22	10
South west	29,594,000	121	30	10
South–south	22,329,000	109	28	10

In order to achieve excellent comparison between income generated by health insurance scheme and the budget per capita of MOH, the revenue generated was calculated per person. The assumption being that on the average, six individuals make up a household. This was the finding from this study and is supported by a similar conclusion in a study done by Fonta, Eme Ichoku and Ataguba [12] while determining WTP among individuals in Nsukka Local government of Nigeria.

For the regional schemes, the South-west regional health insurance scheme generated the highest revenue followed by the South-south regional health insurance scheme. The least revenue was generated by the North-west regional health insurance scheme. Interestingly, the South-south and South-west regional health insurance schemes have the highest TP but the health insurance schemes in the North have the lowest TPs as well as lowest generated revenue.

The urban national insurance scheme generated more funds than the rural national insurance scheme.

Adopting the regional health schemes to cover the whole nation will generate a summative fund of US$13 per individual while a national health scheme will generate a total fund of US$11 per individual per annum. Including the transferred funds of government (budget per capita of MOH) to these schemes will result in total funds generated to be US$23 and US$21 per person per annum for both regional and national schemes respectively.

## Discussion

The mean capacity to pay for the households in different regions ranged from 30,271 ± 15,577 (US$194 ± 100) to 153,558.2 ± 141,306 Naira (US$986 ± 907). There was reduction in the capacity to afford premium as premium increased. This effect was much remarkable in the lowest SES quintile. About sixty percent of the population could afford premium at US$60 annual premium irrespective of the quintile. The threshold premiums of the national health insurance scheme, urban national health insurance and rural health insurance schemes were US$66, US$154 and US$53 respectively.

Invariably, CP reduced significantly across socio economic quintile as revealed in the study. It was not surprising then that affordability of premium, reduced as the CBHI premium increased and the effect was worst on the lowest socioeconomic quintiles. Also, the study showed that at premium above US$60, only 40 % of the population in the lowest socio economic quintile could afford premium. These results confirm the linear relationship between ability to afford premium and socio economic status which agrees with similar findings by some experts [3, 9, 10, 21, 22]. Importantly, this study showed that high premium has worst effect on the first (lowest) quintile implying that adopting higher premium(above US$60) in insurance schemes will lead to low enrolment and disproportionately affect the poorest in the Nigerian society.

The results of different hypothetical schemes considered in this study both at national and regional levels showed that the rural national health scheme appeared to favour the lowest quintile of the population by generating a lower TP which was affordable to 60 % of population in the lowest quintile compared to 48 % for the urban national health insurance scheme.

This allows the conclusion that separating the national health insurance scheme into rural and urban will possibly improve affordability of premium by rural dwellers who are mainly in the lowest socio economic quintile. Most experts [6, 23–25] agree that national or regional health insurance schemes achieve substantial coverage of the population compared to multiple voluntary based community based health insurance schemes.

However, this study does not support adopting TP for regional health insurance schemes because it favours the lowest socio economic groups in some regions at the expense of other regions (for example TP of North-west regional scheme was more affordable to the lowest socio economic quintile compared to South-south regional scheme). This differential effect may be counterproductive if equitable distribution of health services is to be achieved. In this respect, adopting the National rural health insurance scheme whose TP achieves horizontal equity (individuals within the same quintile pay same premium irrespective of their region) across lowest socio economic quintiles in different regions (in Nigeria) may be the best alternative.

From a wider perspective, this study provides evidence that premium generated from WTP studies are lower than the TPs from this study. This confirms earlier claims by some experts [26, 27] that premiums calculated from WTP are not true reflections of what a given population can afford therefore, do not generate optimal revenue for insurance schemes. For instance, the WTP study in the South-east region generated a premium of about US$34.8 [14] whilst TP for a Southeast regional scheme based on this study is US$72.06 (about twice WTP).

The observed difference in premium may be due to the low literacy level among rural dwellers in Nigeria. It might be difficult for them to appreciate the magnitude of their health challenges weighed against other priorities competing for their income. As such, they may not make rational decisions in the choice of what they are willing to pay.

TP determines affordability and not willingness to pay. However, the essence of determining TP is not for policy makers to adopt it as the premium but for premium adopted not to exceed the TP. For instance the premium currently being advocated for CBHI in Nigeria exceeded the TP. If premium is based on WTP and in such cases usually lower than TP, it will be acceptable as long as government is willing to augment it so that the schemes are viable.

With respect to equity, it could be argued that adopting TP as against WTP generated premium may be very regressive to the poor. This was not the case in this study. Adopting hypothetical rural national health insurance scheme showed that about 60 % of the population in the lowest socio economic status (first quintile) were able to afford TP. Yet, TP generated was greater than all premiums generated by WTP studies in Nigeria [12, 14, 17, 28].

From the perspective of financial sustainability of the various health insurance schemes considered, it is quite instrumental to relate the estimate of minimum spending per person per year needed to provide basic lifesaving services as recommended by WHO (US$35–US$50 per person per year) [29] to funds generated by health insurance schemes.

Depending only on budget provision to the ministry of health will imply realising only a third of the recommended minimum spending as revealed by the study. This is further complicated by the fluctuation of fund transfer from the government depending on the priorities of government and the income generated [30]. Consequently, the health sector will be underfunded if it depends solely on government funding. However, including either the regional or national schemes will result in generation of additional funds within the range of US$11–US$13 per person per year. This can only be possible if premium is based on TP and not WTP and also if the health insurance schemes are made compulsory. Most WTP studies [12, 23, 25, 31] conducted in Nigeria generated very low premiums and were based on voluntary membership of the schemes. Implicitly, from the financial perspective, lot of funds will be lost if premium based on WTP were to be used for the health insurance schemes.

However, despite the predicted income that will be generated based on TP, it is still lower than what is recommended by the WHO. Considering that premium predicted is based on maximum premium that can be affordable by the population (if 80 % were to be covered), increasing premium beyond this threshold will exclude the lowest socioeconomic groups from membership of the hypothetical insurance schemes.

Remarkably, the lowest socio economic groups form the bulk of the rural dwellers, as such increasing the premium beyond the TP will result in low enrolment of the rural dwellers. Considering that in Nigeria rural dwellers constitute the bulk of the informal sector [16], it becomes imperative to be discretionary in adopting premium for the informal sector.

However, from the perspective of maximising coverage, it appears more appropriate for policy makers to adopt the scheme that will be affordable for a greater proportion of the least socioeconomic class in the rural areas if premium is based on the predicted TP.

If the interest is to achieve maximal coverage of the rural dwellers then, the rural national health insurance scheme is most appropriate; but if achieving maximal coverage of the informal sector is the priority then a national health insurance scheme will be most ideal.

### Limitations of the study

The limitations of the study was grouped into limitations of the data and limitations of the methods. An observed limitation in the data is the presumed measurement errors in the data collected for non-food expenditure. The data was collected in the post-harvest seasons and no provision was made for repeated collection of data in other parts of the year to allow for calculation of reliability ratios [32].

Considering that income fluctuation is the norm for rural dwellers in the country who are mainly subsistence farmers; Validity of outcome could be improved if data on non-food expenditure during the pre-planting season was available.

Another limitation was that data collection was in 2011, as such; there may be variations in the current capacity to pay of the population due to changes in economic indices. A current data on expenditure on non-food will have improved the validity of the results.

The limitations of method were in the assumptions made in order to model the TPs for the different schemes which may not be perfectly ideal in practice. These include assuming that all households will enrol into the scheme and that government will be willing to cover 80 % of the population. These assumptions will only be feasible if a compulsory scheme is adopted and policy makers are willing to cover 80 % of the population.

Next, was the use of percentile in generating TP which should have high validity if the distribution was perfectly normal. This is usually not the case in population surveys; however to mitigate this, confidence intervals were calculated which did not exceed zero and had a narrow range.

## Conclusions

### Policy implications

The results buttress the opinion of certain authors [15, 33] on the capacity of TP to increase the finances generated by health insurance schemes compared to WTP. It appears that the current national premium being proposed by NHIS exceeded the premiums generated by WTP studies. Unfortunately, the proposed premium for the informal sector national health insurance scheme also exceeded the threshold premium generated in this study (based on a hypothetical national health insurance scheme). The implication being that payment of the NHIS proposed premium by certain individuals in the lowest socio economic class may be regarded as “catastrophic” apparently, in order to avoid untoward effect on membership of the lowest socio economic class, a review of the proposed premium is recommended.

Additionally, the findings in this study that the proportion of lowest socioeconomic group able to afford TP for rural national health insurance scheme was higher than for both National and Urban national health insurance schemes implies that it may be worth considering separating premium of rural social health insurance scheme from that of urban self-employed health insurance scheme such that each scheme has a different premium probably based on the predicted TP from this study considering that a sizeable proportion of the informal sector reside in the rural area.

Finally, policy makers should as a matter of urgency scale up the budget to MOH if universal coverage for basic lifesaving services will be achieved since additional funds based on TP is still not adequate.

### Policy recommendation

The results from this study showed that the capacity to pay reduced across socioeconomic quintiles with the first quintile having the lowest. Also, the proportion of population able to afford premium reduced as premium increased. Effect was worse on the lowest socio economic class. Additionally, the different hypothetical health insurance schemes considered in the study showed that the rural national health insurance scheme and the national insurance schemes generated a favourable TP for the poor rural dwellers. However, the regional schemes generated TPs which were not affordable by a large proportion of population in the lowest socioeconomic quintiles in certain regions. It was also demonstrated in the study that the NHIS proposed premium for the informal sector insurance scheme was above the TP for a national scheme.

Furthermore, it was proven from the study that depending on TP will generate more funds from health schemes than using WTP. Also, both national and regional schemes could not generate sufficient revenue to meet the WHO requirement for basic lifesaving services despite supplementation by MOH budget.

Consequently, the policy recommendations include re-evaluating NHIS proposed premium to reflect the threshold premium; since the current premium exceeded TP as well as increasing budgetary allocation to ministry of health, in order to achieve a financially viable health insurance scheme.

## Abbreviations

TP: threshold premium
CBHI: community based health insurance
CP: capacity to pay
WTP: willingness to pay
MOH: Ministry of Health
NHIS: national health insurance scheme
